# Interaction of Programmed Obesity and Postnatal High Fat Diet but Not (-)-Epicatechin Treatment Modifies Muscle Atrophy Proteins Levels in Male Wistar Rats

**DOI:** 10.1007/s12013-025-01690-w

**Published:** 2025-03-15

**Authors:** Ana Luisa Alvarez-Chávez, Sergio De los Santos, Ramón Mauricio Coral-Vázquez, Luis Antonio Reyes-Castro, Elena Zambrano, Patricia Canto

**Affiliations:** 1 Obesity Research Unity, Medicine Faculty, Mexico Autonomous National University, Mexico City, México; 2 Clinical Research Subdirection, Research Direction, National Institute of Medical Sciences and Nutrition “Salvador Zubirán”, Mexico City, México; 3 Postgraduate Studies and Research Section, Higher School of Medicine, National Polytechnic Institute, Mexico City, México; 4 Subdirection of Teaching and Research, National Medical Center “20 de Noviembre”, Institute of Security and Social Services of State Workers, Mexico City, México; 5 Reproduction Biology Department, National Institute of Medical Sciences and Nutrition “Salvador Zubirán”, Mexico City, México; 6 Faculty of Chemistry, Mexico Autonomous National University, Mexico City, México

**Keywords:** Obesity by fetal programming, Obesity by doble stimulus, Gastrocnemius and soleus muscle, Proteins synthesis and muscle atrophy, (-)-epicatechin

## Abstract

We determine whether the offspring of obese mothers and a postnatal high-fat diet (HFD) modify protein levels related to muscle synthesis (p70S6K-alpha) or atrophy (Murf and MAFbx), and if the administration of (-)-epicatechin (Epi) can modify these alterations. We hypothesized that the ubiquitin ligases Murf and MAFbx would be increased in the obesogenic context, either by in utero obesogenic environment or by a postnatal high-fat diet, while the p70S6K-alpha kinase and its activation might be decreased. Eight groups of six male Wistar offspring formed eight experimental groups: control (C), control fed with HFD (CHFD), maternal obesity (MO), maternal obesity fed with HFD (MOHFD), and the groups with Epi intervention: C+Epi long, CHFD+Epi long, MO+Epi long and MOHFD+Epi long. By Western blot, we evaluated the Epi effect on the Murf, MAFbx, and p70S6K-alpha proteins in gastrocnemius and soleus tissues. The Murf level increased 2.59-fold in CHFD vs C group and 2.62-fold for MOHFD vs C group (*p* = 0.049 and *p* = 0.048, respectively) in gastrocnemius tissue. In soleus tissue, we observed an increase of MAFbx (1.52-fold) for the MOHFD group versus the C group (*p* = 0.049). Epi treatment did not modify any protein expression. In conclusion, we found an increase in the Murf1 protein levels in gastrocnemius tissue of the direct model of obesity; as well, we observed an increase of the Murf1 in gastrocnemius and of the MAFbx in soleus muscles in the group of rats obese by programming and fed postnatally with a high-fat diet (doble stimulus). In addition, since obesity could cause muscle atrophy, which results in impaired muscle function, it would be relevant in future research to evaluate these signaling pathways in animals of different ages in order to search for markers of the progression of diseases such as sarcopenia obesity.

## Introduction

Maternal obesity is considered a condition of fetal programming for obesity. This phenomenon has received special attention in recent years due to the increase in obesity in women of reproductive age [1]. This is important, as maternal obesity has a detrimental effect on the diverse tissues of the offspring [2]. Besides, it is worrying that the damaging impact on the health of the offspring with obesity due to programming can last a lifetime, especially when combined with a harmful environment in adulthood, such as the consumption of a high-fat diet (HFD) [2].

Skeletal muscle (SM) is one of the main tissues affected by obesity by programming, which can present atrophy, reduction of muscle fibers with an accumulation of lipids intramuscular [3], an increase of immune cell infiltration [4], a diminished of the lean mass [5] and fewer total nuclei *per* fiber [6], among others. Likewise, the offspring of obese mothers fed postnatally with a high-fat diet (HFD) also present a dysfunctional SM, as impairments in muscle regeneration, reduced gene expression levels of postnatal myogenesis markers [6], and decreased lean mass [7].

Sishi et al., [8] have described that high-fat diet obese rats presented gastrocnemius muscle atrophy. One of the signalling pathways involved in the pathogenesis of this phenomenon is the ubiquitin-proteasome pathway, specifically the ubiquitin-protein ligases muscle ring finger protein 1 (Murf1) and muscle atrophy F-box (MAFbx). Both ubiquitins are overexpressed through a different trigger, the NF-κB and MAPK pathways for Murf1 and MAFbx, respectively.

Regarding muscle protein synthesis, the phosphorylation of the kinase p70S6K-alpha via mTOR participates in the development of muscle fibers [9]. It has been described in obese offspring by programming a decrease in the expression of this protein compared to the controls [4].

On the other hand, several nutraceutical derivatives are being studied to evaluate their beneficial effects on obesity and the reduction of its comorbidities, among which is the flavonoid (-)-epicatechin (Epi), a compound found in various foods such as cocoa, tea, and red fruits [10]. Notably, it has also shown the beneficial effects of Epi over muscle atrophy through several mechanisms [11–13].

Furthermore, our research group demonstrated that obesity by programming due to maternal obesity, coupled with postnatal HFD feeding, decreased lean mass, and the postnatal administration of Epi, in both models of obesity, could increase the lean mass in these offspring [5, 7].

Although programmed obesity decreases lean mass, and the flavonoid (-)-epicatechin administration may increase it [5, 7], to our knowledge, no possible molecular pathways have been described that participate in both conditions. Therefore, the present study aimed to examine whether offsprings of obese mothers fed postnatally with a control diet or a high-fat diet present variations in protein levels related to gastrocnemius and soleus muscle atrophy and whether administration of the flavonoid (-)-epicatechin can modify these alterations. We hypothesized that the ubiquitin ligases Murf and MAFbx will be increased in the obesogenic context, either by *in utero* obesogenic environment or by postnatal high fat diet, while the p70S6K-alpha kinase and its activation might be decreased.

## Materials and Methods

### Animal Model

The rodent studies complied with the ARRIVE guidelines and followed the U.K. Animals (Scientific Procedures) Act, 1986 and associated guidelines, as well as the Laboratory Animal Resources Institute’s Guidelines for the Care and Use of Laboratory Animals (http://www.nal.usda.gov/awic/pubs/noawicpubs/careuse.htm) and by the Official Mexican Standard NOM-062-ZOO-1999. Besides, the protocol was authorized by the Committee of Ethics in Animal Experimentation of the National Institute of Medical Sciences and Nutrition Salvador Zubirán (CICUAL-UIO-2105-23-25-1).

In this study, we analyzed Wistar rats (*Rattus norvegicus*, Taxonomy ID: 10116). Previously, our research group provided comprehensive details of the standardization of the female phenotype (F0) employed for generating the F1 offspring, the Chow and HFD composition, and the 13-week (-)-epicatechin intervention [5]. Briefly, at postnatal day 21, from each litter, one female albino Wistar rat was randomly selected to be assigned to the control group (C, *n* = 6, laboratory Chow diet) or the maternal obesity group (MO, *n* = 6, fed with an HFD, energy 4.9 kcal/g).

**Fig. 1:**
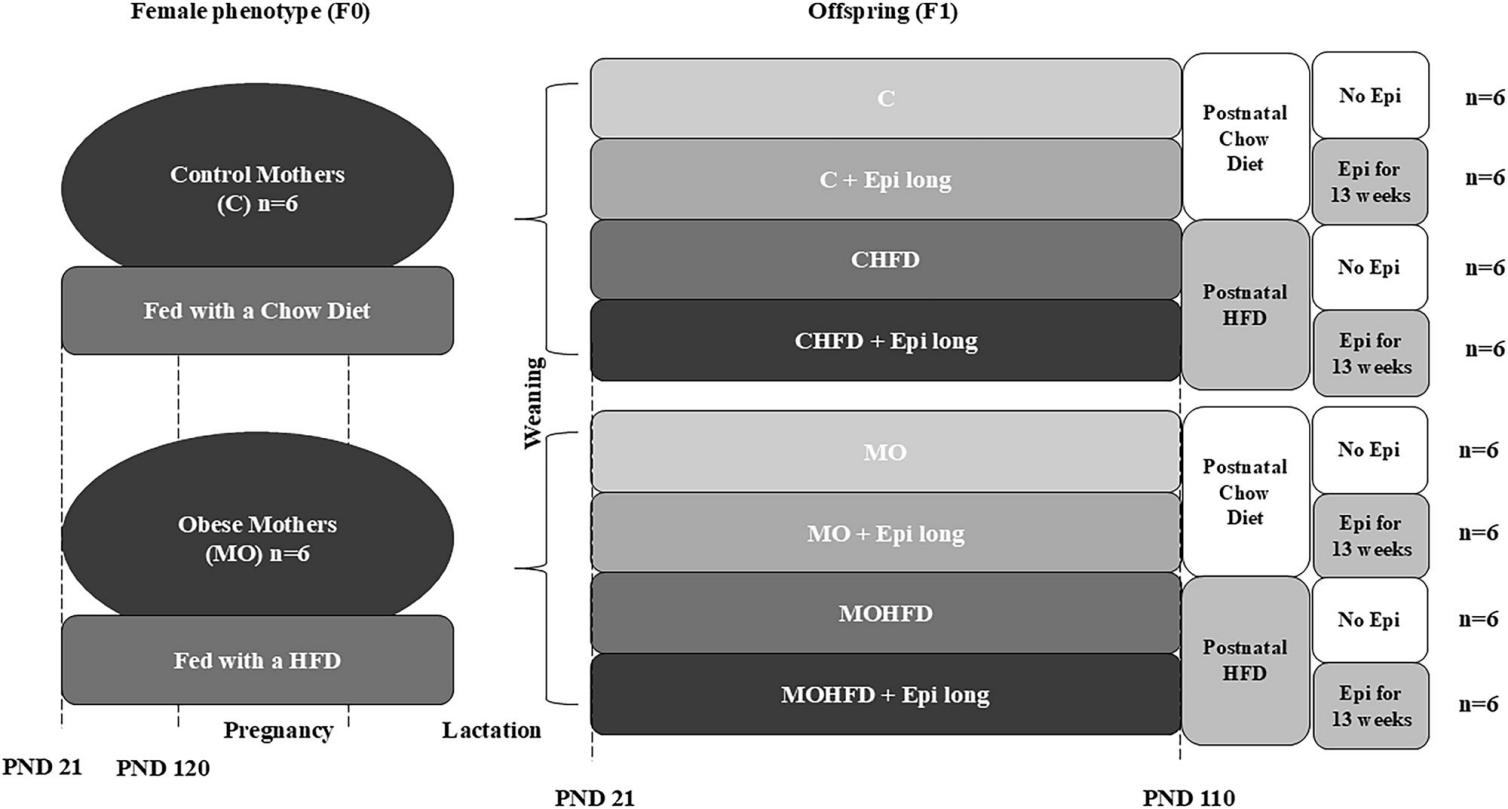
Design of the study. Six female rats (F0) for each group (C or MO) were fed with control or high-fat diet from weaning and during pregnancy and lactation. At postnatal day 21, 24 males per group (C and MO) from different litters were randomly allocated to be fed control or HFD and treated with water or with (-)-epicatechin twice *per day* for 13 weeks (C, C + Epi long, CHFD, CHFD + Epi long, MO, MO + Epi long, MOHFD, MOHFD + Epi long, *n* = 6 rats *per* group)

The twelve F0 female rats were mated with tested male breeders at 120 postnatal days (pnd) and conceived during the subsequent cycle. During pregnancy and lactation, all Wistar F0 albino rats maintained their respective diets (Chow or HFD) (Fig. 1).

The F1 progeny underwent weaning on pnd 21 and were subjected to either standard chow (C) or HFD throughout the study period until pnd 110. (-)-Epicatechin intervention was implemented from day 21–110 pnd (13 weeks of long Epi treatment) [7, 14]. Male pups were administered either water (as a vehicle) or 1 mg/kg body mass of Epi (Sigma-Aldrich, St Louis, MO, U.S.A.) via oral catheter twice a day [5, 7]. The male offspring (F1) from either the control or maternal obesity group, from different litters, were randomly allocated to six experimental groups for the analysis of gastrocnemius muscle: offspring of control group (C, *n* = 6), offspring of control group fed with HFD (CHFD, *n* = 6), offspring of control group fed with HFD treatment with Epi (CHFD + Epi long, *n* = 6), offspring of maternal obesity group (MO, *n* = 6), offspring of maternal obesity group fed with HFD (MOHFD, *n* = 6), offspring of maternal obesity group fed with HFD treatment with Epi (MOHFD + Epi long, *n* = 6).

For the soleus muscle, we formed eight experimental groups: offspring of control group (C, *n* = 6), offspring of control group treatment with Epi (C + Epi long, *n* = 6), offspring of control group fed with HFD (CHFD, *n* = 6), offspring of control group fed with HFD treatment with Epi (CHFD + Epi long, *n* = 6), offspring of maternal obesity group (MO, *n* = 6), offspring of maternal obesity group treatment with Epi (MO + Epi long, *n* = 6), offspring of maternal obesity group fed with HFD (MOHFD, *n* = 6), offspring of maternal obesity group fed with HFD treatment with Epi (MOHFD + Epi long, *n* = 6). We analysed only six groups for gastrocnemius muscle because the C + Epi long and MO + Epi long groups were described in a previous manuscript [15].

At postnatal day 110, after 6 h of fasting, rats were anaesthetized with isoflurane (based on the instructions for the use of the isoflurane inhaler of the brand: Stoelting Co, Cat. No. 50207 regarding animal weight) and euthanized followed by isolation of the soleus and gastrocnemius muscles by a trained person and following the reported protocol [16].

### Western Blotting

The proteins of the gastrocnemius and soleus muscles in the eight experimental groups were analyzed by Western Blott. Around 100 mg of tissue was homogenized in RIPA buffer (Santa Cruz Biotechnology, Inc., USA) along with protease and phosphatase inhibitors. We performed one replicate *per* animal, having six animals per group (*n* = 6). For each sample, 50 μg of protein was run on an SDS-PAGE gel and transferred to a nitrocellulose membrane (Pierce, P01-88018, Germany). Subsequently, the membranes were incubated with the different primary antibodies: recombinant Anti-MURF1+MURF3+MURF2 (ab172479, Abcam, United Kingdom); Recombinant anti-Fbx32 (ab168372, Abcam, United Kingdom); anti-ribosomal protein S6 kinase beta-1 (p70S6K-alpha) (49D7, rabbit mAb #2708, Cell Signaling, USA); anti-Phospho-p70 S6 kinase (Thr389) (108D2, Rabbit mAb #9234, Cell Signaling, USA); and anti-Gadph (PA1-988, ThermoFisher Scientific, USA). After the incubation with the primary antibody. Then the membranes underwent an incubation with the appropriate secondary antibody: HRP-goat anti-rabbit IgG (111-095-003) or HRP- anti-mouse IgG (715-035-150) (Jackson Inmuno Research Laboratories, I.N.C., U.S.A.). In order to detect the protein level, the SuperSignal^tm^ West Femto Chemiluminescent Substrate kit (ThermoFisher Scientific, U.S.A.) was used. We used the protein GAPDH as loading control and the quantification results are expressed as the ratio between the interest protein and the GAPDH density in the membrane after performing the Western Blot technique.

Detection of band intensities was digitally quantified using LI-COR Image Studio software (http://www.licor.com/bio/products/software/image_studio_lite/) and ImageJ software (U. S. National Institutes of Health, Bethesda, Maryland, U.S.A.).

### Statistical Analysis

The normal distribution of the data was analyzed by the Shapiro-Wilk test, fulfilling the normality criteria, along with the assumption of variance homogeneity evaluated by the Brown-Forsythe test. Under these conditions, we decided to perform parametric tests in every analysis. Data are expressed as mean ± standard deviation of six individual experimental observations analyzed with GraphPad Prism 6.0 software (GraphPad Software, San Diego, CA). As described previously, we worked with six experimental gastrocnemius muscle groups. This allowed us to identify the differences between specific groups by doing a one-way ANOVA followed by a Tukey post hoc test. To analyze the protein level of the eight experimental groups in soleus muscle, since we had a factorial design of three variables, a three-way ANOVA was performed to determine the influence of each of the three variables over the model (maternal diet, postnatal diet, and Epi treatment) and possible interactions between them, followed by a one-way ANOVA and a Tukey *post hoc* test to determine differences between individual groups. The three-way ANOVA could only be performed in the soleus factorial design. However, comparing groups allowed us to get valuable information from the gastrocnemius and soleus muscles. Significant differences were defined by a *p* < 0.05.

## Results

**Fig. 2:**
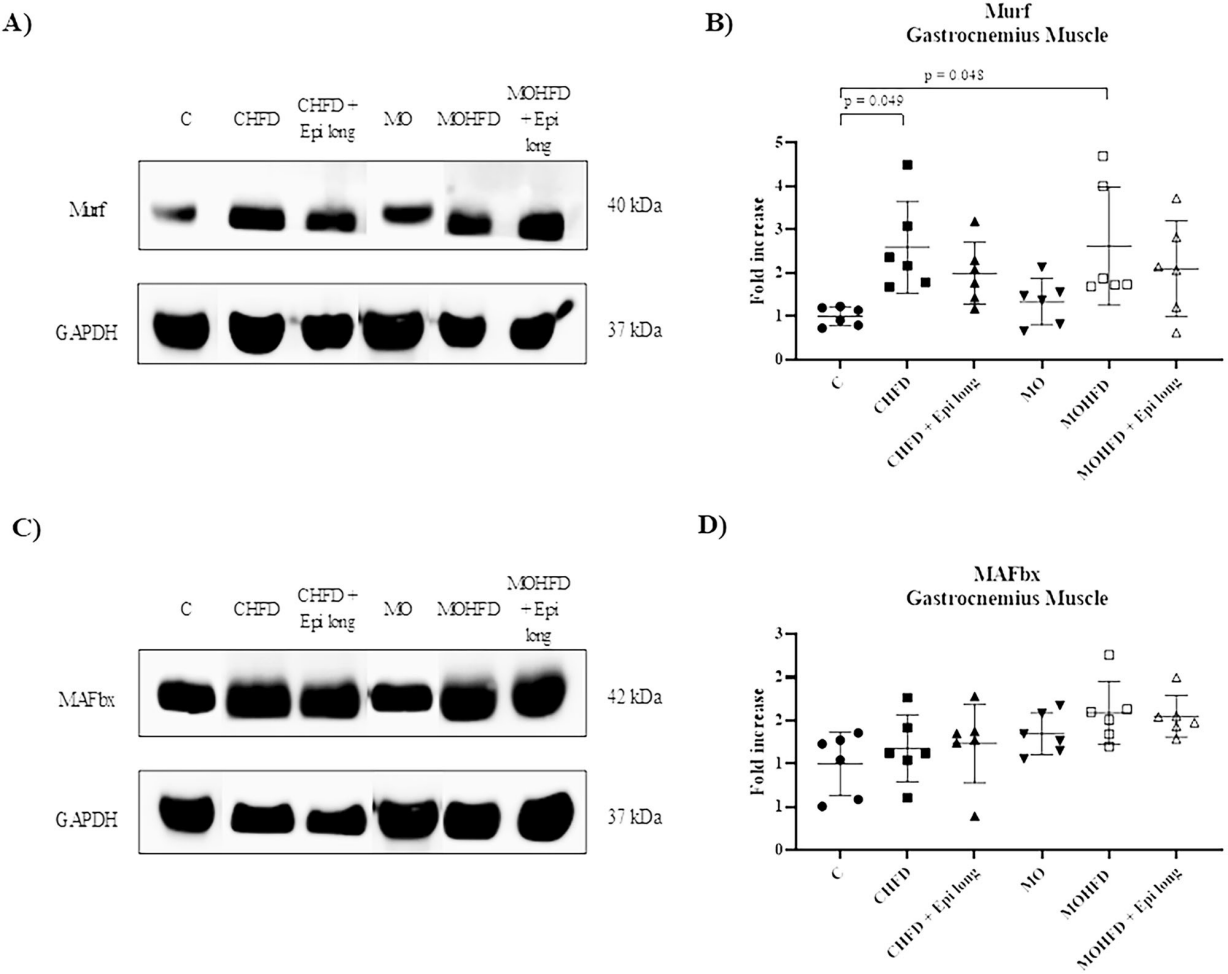
Effect of postnatal high-fat diet and Epi treatment for 13 weeks on ubiquitin ligases expression in gastrocnemius muscle of male offspring of control or obese mothers at 110 postnatal days. **A**) and **C**) Representative immunoblotting of proteins Murf and MAFbx. GAPDH was used as a loading control. **B**) and **D**) Densitometry analysis of Murf and MAFbx protein expression in gastrocnemius muscle. The HFD in the C and MO group (CHFD and MOHFD) increased the Murf1 protein levels (*p* = 0.049 and *p* = 0.048, respectively) (Fig. 2, Panel B). Data are expressed as median and standard deviation and were analyzed by a one-way ANOVA followed by *post hoc* Tukey Test for pair comparison (*n* = 6 rats *per* group). C male rats offspring of control mothers, CHFD male rats offspring of control mothers with postnatal high-fat diet, CHFD + Epi long male rats offspring of control mothers with postnatal high-fat diet and treatment with Epi for 13 weeks, MO male rats offspring of obese mothers, MOHFD male rats offspring of obese mothers with postnatal high-fat diet, MOHFD + Epi long male rats offspring of obese mothers with postnatal high-fat diet and treatment with Epi for 13 weeks

Regarding the gastrocnemius tissue analysis, we observed that the Murf protein levels increased 2.59-fold in the CHFD vs. C group and 2.62-fold for MOHFD compared to the C group (*p* = 0.049 and *p* = 0.048, respectively) (Fig. 2A, B). Still, we only observed a trend of increasing MAFbx protein levels in the MOHFD group *versus* the control group (*p* = 0.067) (Fig. 2C, D). However, Epi treatment did not modify the Murf or MAFbx protein levels in any experimental groups (CHFD+Epi long and MOHFD+Epi long) (Fig. 2A–D).

**Fig. 3:**
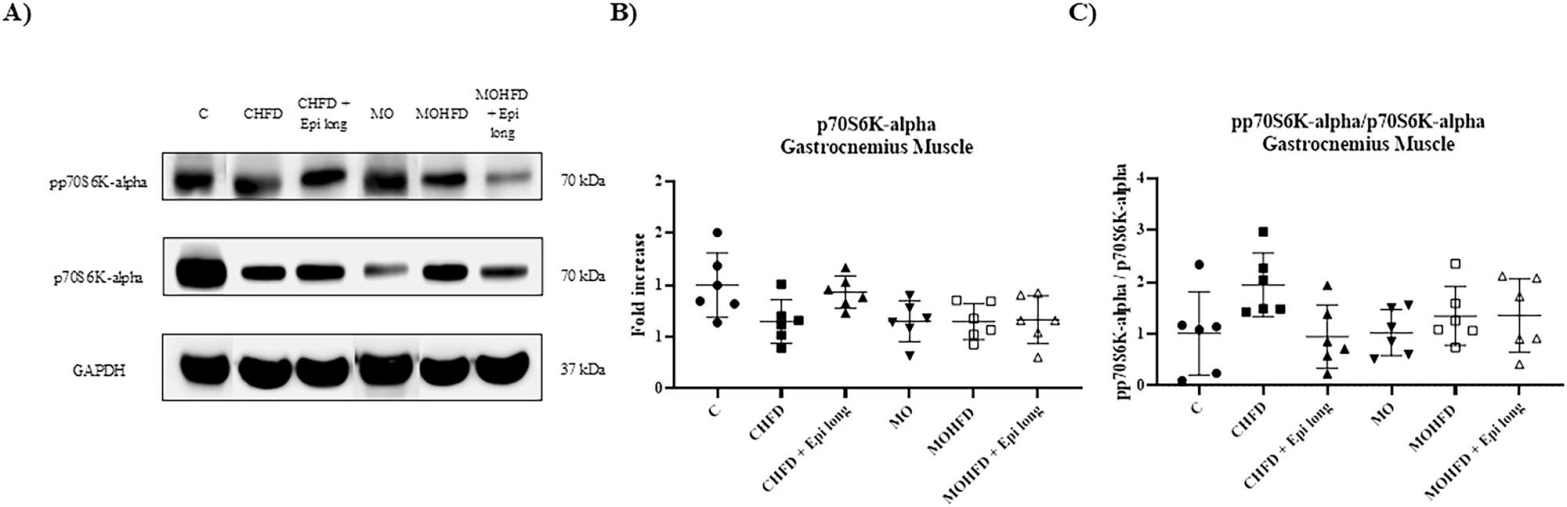
Effect of postnatal high-fat diet and Epi treatment for 13 weeks on protein synthesis in gastrocnemius muscle of male offspring of control or obese mothers at 110 postnatal days. **A**) Representative immunoblotting of p70 S6K-alpha total and phosphorylated protein. GAPDH was used as a loading control. Densitometry analysis of protein expression **B**) and **C**) p70 S6K-alpha total and the ratio between phosphorylated and total p70 S6K-alpha in gastrocnemius muscle. Data are expressed as median and standard deviation and were analyzed by a one-way ANOVA followed by *post hoc* Tukey Test for pair comparison (*n* = 6 rats *per* group). C male rats offspring of control mothers, CHFD male rats offspring of control mothers with postnatal high-fat diet, CHFD + Epi long male rats offspring of control mothers with postnatal high-fat diet and treatment with Epi for 13 weeks, MO male rats offspring of obese mothers, MOHFD male rats offspring of obese mothers with postnatal high-fat diet, MOHFD + Epi long male rats offspring of obese mothers with postnatal high-fat diet and treatment with Epi for 13 weeks

To evaluate the influence of a postnatal high-fat diet and Epi treatment on muscle protein synthesis, we determined the expression of total p70S6K-alpha and its phosphorylated form. Still, we found no significant differences between them in the experimental groups (Fig. 3A–C).

**Fig. 4:**
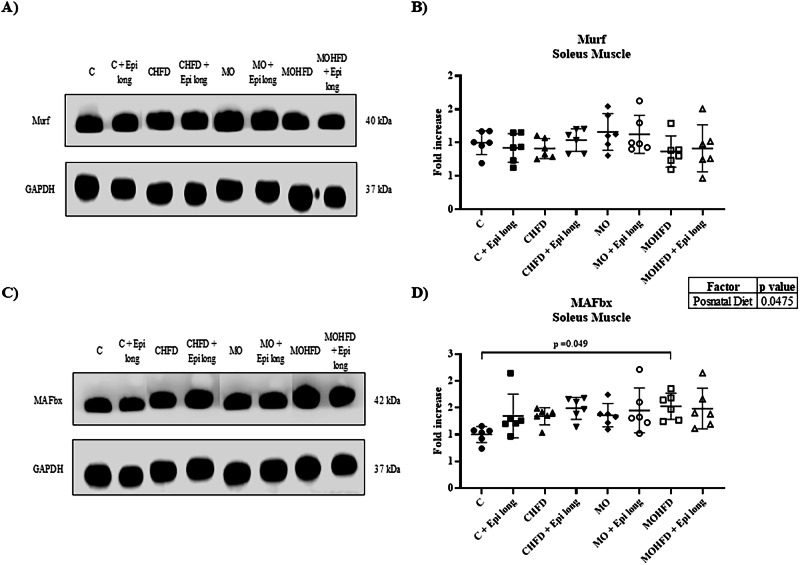
Effect of postnatal high-fat diet and Epi treatment for 13 weeks on ubiquitin ligases expression in soleus muscle of male offspring of control or obese mothers at 110 postnatal days. **A**) and **C**) Representative immunoblotting of proteins Murf and MAFbx. GAPDH was used as a loading control. **B**) and **D**) Densitometry analysis of Murf and MAFbx protein expression in soleus muscle. Data are expressed as median and standard deviation and were analyzed by a three-way ANOVA, afterward analyzed by a one-way ANOVA followed by *post hoc* Tukey Test for pair comparison (*n* = 6 rats *per* group). C male rats offspring of control mothers, C + Epi long male rats offspring of control mothers postnatally treated with Epi for 13 weeks CHFD male rats offspring of control mothers with postnatal high-fat diet, CHFD + Epi long male rats offspring of control mothers with postnatal high-fat diet and treatment with Epi for 13 weeks, MO male rats offspring of obese mothers, MOHFD male rats offspring of obese mothers with postnatal high-fat diet, MOHFD + Epi long male rats offspring of obese mothers with postnatal high-fat diet and treatment with Epi for 13 weeks

Moreover, regarding the results obtained of the MAFbx expression in soleus tissue, after performing the three-way ANOVA analysis, we observed a significant contribution of the postnatal diet to the overall variation of the model (Postnatal diet 7.76% of the variation, *p* = 0.047) (Fig. 4C, D; Table 1). Remarkably, after performing the multiple comparison analysis, we found a significant increase of MAFbx (1.52-fold) for the MOHFD group compared with the C group (*p* = 0.049); however, Epi treatment did not modify the expression of this ligase (Fig. 4C, D). In addition, the level of Murf protein in the eight experimental groups in soleus tissue did not change (Fig. 4A, B), nor did the three variables (maternal diet, postnatal diet, or treatment with Epi) interact in the model (Fig. 4A, B).

**Table 1:** Three-way ANOVA analysis of the Soleus muscle of the male offspring obese by programming and/or postnatally fed with high-fat diet and with (-)-epicatechin treatment

	Maternal diet	Offspring diet	Epi treatment
	C *vs*. MO groups	Postnatal chow diet *vs*. Postnatal HFD diet	No Epi *vs*. Epi long
Murf	F = 0.489 *p* = 0.488	F = 2.944 *p* = 0.094	F = 0.043 *p* = 0.836
MAFbx	F = 3.605 *p* = 0.065	F = 4.181 *p* = 0.047	F = 2.548 *p* = 0.118
p70S6K	F = 0.168 *p* = 0.684	F = 0.095 *p* = 0.760	F = 0.233 *p* = 0.631
pp70S6K	F = 0.015 *p* = 0.902	F = 0.376 *p* = 0.543	F = 0.009 *p* = 0.935

*Epi* (-)-epicatechin, *C* male rats offspring of control mothers, *MO* male rats offspring of obese mothers, *HFD* high fat diet, *Murf* muscle ring finger protein 1, *MAFbx* muscle atrophy F-box, *p70S6K* ribosomal protein S6 kinase beta-1, *pp70S6K* phospho ribosomal protein S6 kinase beta-1

**Fig. 5:**
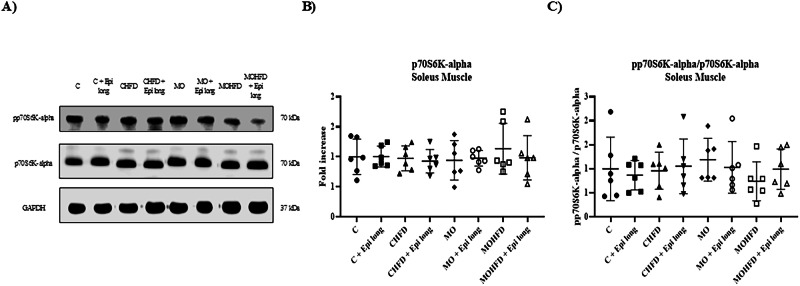
Effect of postnatal high-fat diet and Epi treatment for 13 weeks on proteins related to atrophy and synthesis of proteins in soleus muscle of male offspring of control or obese mothers at 110 postnatal days. **A**) Representative immunoblotting of p70 S6K-alpha total and phosphorylated protein. GAPDH was used as a loading control. Densitometry analysis of protein expression **B**) and **C**) p70 S6K-alpha total and the ratio between phosphorylated and total p70 S6K-alpha in soleus muscle. Data are expressed as median and standard deviation and were analyzed by a three-way ANOVA, afterward analyzed by a one-way ANOVA followed by *post hoc* Tukey Test for pair comparison (*n* = 6 rats *per* group). C male rats offspring of control mothers, C + Epi long male rats offspring of control mothers postnatally treated with Epi for 13 weeks CHFD male rats offspring of control mothers with postnatal high-fat diet, CHFD + Epi long male rats offspring of control mothers with postnatal high-fat diet and treatment with Epi for 13 weeks, MO male rats offspring of obese mothers, MOHFD male rats offspring of obese mothers with postnatal high-fat diet, MOHFD + Epi long male rats offspring of obese mothers with postnatal high-fat diet and treatment with Epi for 13 weeks

Finally, we analyzed the total and phosphorylated expression of p70S6K-alpha (a kinase related to the synthesis of musculoskeletal proteins) but did not demonstrate changes in any of the experimental groups. There were also no interactions between the three variables (maternal diet, postnatal diet, or treatment with Epi) throughout the model (Fig. 5A–C).

## Discussion

Obesity caused by a high-fat diet is associated with dysfunction of the skeletal muscle characterized by reduced myofiber, satellite cells, and skeletal muscle mass, among other changes. These changes lead to a morphological change that results in an atrophic phenotype [17–19]; on these bases, HFD is a risk factor for muscle atrophy.

De los Santos et al. [5] described that male rat Wistar with obesity by programming presented a decrease in lean mass, and the administration of the flavonoid (-)-epicatechin could increase it. In addition, in 2022, the same research group showed that rats obese by programming fed postnatally with a high-fat diet too presented a decrease in lean mass when compared to a direct obesity model and its controls, and the (-)-epicatechin treatment improved the body composition [7]. Although, these studies did not analyze the possible molecular mechanisms involved in these alterations. On these bases, this study aimed to analyze if the offspring of obese mothers present disorders in the expression of genes related to atrophy or protein synthesis in the gastrocnemius and soleus muscles and if a high-fat postnatal diet also modifies these alterations, as well as by the administration of the flavonoid (-)-epicatechin. It is important to note that previously, we confirmed that both models (direct obesity and obesity by programming) develop obesity [5, 7].

Skeletal muscle morphology and function depend on muscular protein turnover in which the anabolism and catabolism processes are tightly regulated. The PI3K/Akt/mTOR signalling cascade is the main anabolic pathway, and it comprises several downstream cascades that could affect the skeletal muscle protein synthesis [20, 21], including the regulation of other molecules associated with protein degradation. The ubiquitin ligases MURF and MAFbx are the most described and best-understood mediators of muscle protein degradation through the ubiquitin-proteasome system. They are specific for diverse muscle proteins ranging from components of the contractile machinery to molecules related to myoblast differentiation [21].

Several studies have analyzed in a direct obesity model by HFD, the expression of MAFbx, Murf1 proteins in the gastrocnemius muscle of male rodents (mice and Sprague-Dawley rats) obese by HFD finding an increase in the expression of these ubiquitin ligases [18, 19, 22, 23]. The mechanism by which this deleterious stimuli induced an increase in the ubiquitin ligase expression is mediated by the decrease in the PI3K/AKT activation, that leads to a decrease in the FOXO phosphorylation, inducing its traslocation to the nucleus, where it acts as transcription factor for the ubiquitin ligases Murf an MAFbx [20], Also, the increased transcription of Murf could be as consequence of the activation of NFκB transcription factor by proinflammatory cytokines, that are increased in the context of obesity [8].

Regarding our results, we only observed a significant increase of the Murf1 protein in the gastrocnemius muscle of the direct model of obesity (CHFD group), according to the findings reported by Sishi et al. [8] in male rat Wistar obese by HFD. The latter could be because a direct model of obesity by an HFD is a factor of greater weight than the obesity by programming in the expression of the ligases related to muscle atrophy like it has been suggested for the accumulation of fat and resistance to insulin in the same model [24].

Interestingly, we also observed an increase of the Murf1 and MAFbx in gastrocnemius and soleus muscles, respectively, in the rats obese by programming and fed postnatally with a high-fat diet (MOHFD group). To our knowledge, there are no reports on the effect of the combination of obesity by programming and feeding after weaning in this offspring with HFD in the expression of both leagues in the soleus and/or gastrocnemius muscles. The muscle atrophy secondary to an HFD affects the fiber type in a specific form and depends on the duration that the models received this diet; so, a short administration (3–4 weeks) impacts the oxidative slow-twitch (type I) muscles, like soleus muscle [25, 26], but not type II fiber (glycolytic fast-twitch), as an extensor digitorum longus and gastrocnemius muscles [16]. Interestingly, in our study, even though we give the HFD administration for a long time (~13 weeks), we observed an increase in the expression of the MAFbx in the soleus muscle and of Murf1 in gastrocnemius muscle in the group of rats that had a double stimulus (obese by programming and fed postnatally with a high-fat diet). This could be because it has been described that there is a shift from type I oxidative myofibers to type IIB-IIX glycolytic myofibers as a consequence of obesity [26–28]. Another explanation as the cause of the increase of the expression of both ligases in the MOHFD group is the additive effect between these two deleterious stimuli on the atrophied muscle, as described by Simar et al. [29] regarding the negative impact that obesity by programming plus an HFD postnatal on skeletal muscle markers of glucose and lipid metabolism.

Our findings show that Murf1 is increased after a postnatal HFD, in the control and maternal obesity background, but only in gastrocnemius muscle, while MAFbx is increased in the MOHFD group in the soleus muscle. This evidence raises questions regarding the specificity of these proteins across muscle fibers and atrophic scenarios. Some studies have demonstrated that the increase in the ubiquitin ligases is predominantly fast compared to slow-twitch fibres [30, 31]; besides, other studies have described that under the same stimulus, only one of the two ubiquitin ligases is increased, but not both [8, 32]. The differences might be related to the target proteins of these ligases, Murf’s target proteins are mainly myofibrillar proteins, like nebulin, titin, myosin-binding protein C and myosin light and heavy chains [33], and its action might be more important in the postnatal period and thereby mostly affected by postnatal stimulus, when organisms have more contractile machinery protein turnover; also, Murf has been described to target enzymes involved in the generation of ATP; especially involved in glycolysis [34], which might explain why it might be more present in glycolytic muscle type.

On the other hand, the target proteins for MAFbx are the myogenic regulatory factor MyoD, and eukaryotic translation initiation factor 3 subunit f (eIF3-f) [33], which could be more relevant in the embryogenic period, when abundant cellular differentiation takes place and could explain why MAFbx is particularly affected by both in utero and postnatal deleterious stimuli. Besides, it has been described that the oxidative muscle fibres have more satellite cells, compared to the glycolytic ones [35], and could be more abundant in differentiation factors like MyoD, which could help explain why the soleus muscle shows an increase in MAfbx but nut the gastrocnemius. Nevertheless, additional studies are needed to address the differences between the ubiquitin ligases expression in distinctive muscle fibers types and atrophic conditions.

Regarding the impact on musculoskeletal health, we believe that direct obesity could cause damage to the muscle earlier than programmed obesity, but both conditions have a deleterious effect

Due to the increase in the expression of Murf1 and MAFbx in the skeletal muscle in the group of rats that presented direct obesity or secondary to a double stimulus, we probe if the treatment with the flavonoid Epi could diminish the increase in the expression of these ubiquitin ligases, and thereby prevent muscle atrophy. The latter is because it has been described that this flavonoid is capable in an in vitro model of atrophy [36] and in vivo models [37, 38] of mitigating muscle atrophy. Nevertheless, we did not observe that (-)-epicatechin treatment modified the expression of Murf1 and MAFbx in the skeletal muscle. Our results are like a report on an in vitro model of atrophy, in which Epi does not modify the expression of both ubiquitin ligases in C2C12 myotubes [36, 39]. Although Gonzalez-Ruiz et al. [37] showed that Epi was able to decrease the expression of Murf1 and MAFbx of skeletal muscle (gastrocnemius-soleus) of Long Evans rats, these rats are a model of atrophy by spinal cord injury and not present obesity. They are older than our young rats.

**Fig. 6:**
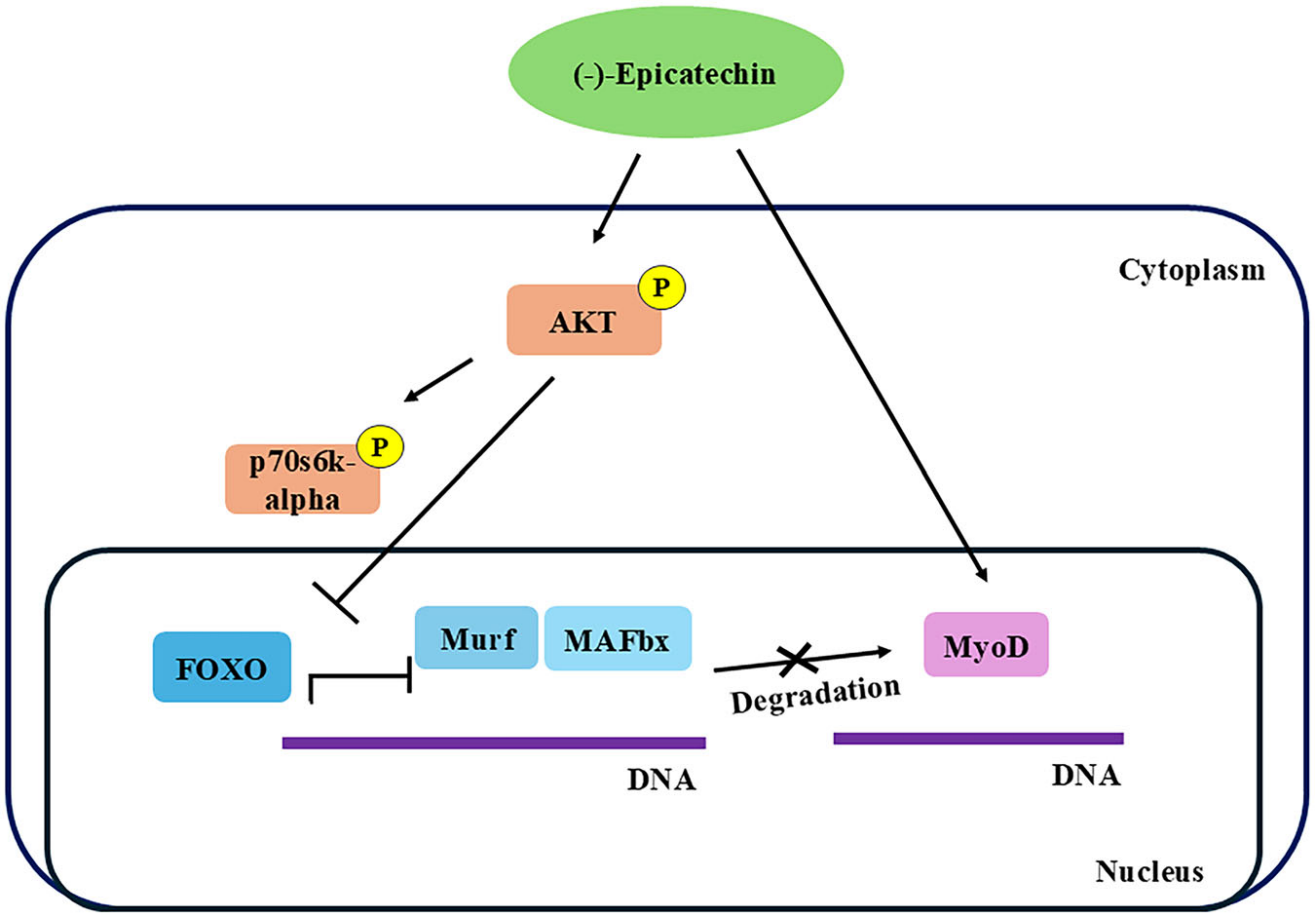
Mechanism by which (-)-epicatechin exerts beneficial effects over skeletal muscle tissue. (-)-epicatechin promotes the activation of the PI3K/Akt signaling pathway that induces the phosphorylation via mTOR of the kinase p70S6K-alpha, related with the synthesis of skeletal muscle proteins. On the other hand, the activation of Akt induces the phosphorylation of FOXO, a transcription factor that when being added a phosphate group, traslocates outside of the nucleus, unable to act as a transcription factor for the ubiquitin ligases MURF and MAFbx, therefore decreasing the degradation of skeletal muscle protein via the ubiquitin-proteosome pathway. Finally, this flavonoid induces the expression of MyoD, transcription factor related to the differentiation of skeletal muscle myofibers

On the other hand, we did not find differences in the expression of the p70s6k-alfa kinase in the gastrocnemius or soleus muscle in any of the experimental groups. It has been described that the activation of this molecule via the addition of phosphate groups exerts a positive effect on the synthesis of musculoskeletal proteins and in the process of maintaining a proper muscular fiber size [20]. Contrary to our results, Arunkumar and Anuradha [40] demonstrated in a murine model that a high-fat diet induces a decrease in the phosphorylation of the protein in skeletal muscle; however, Brown et al. [41] have shown that after a high-fat diet, there is no change in the pp70S6K/p70S6K ratio. In addition, Epi treatment did not modify the pp70S6K in the soleus or gastrocnemius muscles. Therefore, we hypothesize that Epi treatment could act through different molecular pathways in skeletal muscle due to the distinct genetic regulation of this tissue in response to an obesogenic environment (Fig. 6). In this sense, a previous study reported that Epi modulates the activity of antioxidant molecular pathways, increasing its activity [42]. This effect may be relevant to ameliorate the influence of obesity on muscle atrophy. Furthermore, Epi treatment has been shown to reverse sarcopenic effects in rat models, improving regulatory pathways that increase skeletal muscle mass and function [43], exerting a therapeutic effect by accelerating regulatory repair pathways in skeletal muscle after BaCl2-induced damage [38], and increasing the gene expression of myomiRNAs involved in the adaptation of skeletal muscles to exercise accompanied by muscle hypertrophy and an increase in myogenic proteins MyoD and myogenin levels [44].

This study has some limitations. One is that we cannot carry out the histopathological analysis to confirm whether the rats with obesity by programming or with obesity secondary to a doble stimulus had muscle atrophy. Another limitation is that we did not explore another pathway related to protein synthesis. Besides, the fact that we eliminated two groups in the gastrocnemius analysis prevented us from having a factorial design that a three-way ANOVA could evaluate, as we did for the soleus muscle, so we were not able to describe the contribution of each variable to the total variability of the model. However, since we performed a one-way ANOVA with the post hoc test for the gastrocnemius muscle, we were still able to identify the differences between specific groups and the impact of the postnatal high-fat diet by comparing the groups with this diet versus their respective controls (C vs CHFD and MO vs MOHFD), along with the impact of the Epi treatment over the experimental groups with HFD (CHFD vs CHFD + Epi long and MOHFD vs MOHFD + Epi long).

In conclusion, we found an increase in the Murf protein levels in gastrocnemius tissue of the direct model of obesity. Further, we observed an increase of the Murf1 in gastrocnemius and of the MAFbx in soleus muscles in the group of rats obese by programming and fed postnatally with a high-fat diet (doble stimulus). We did not demonstrate that flavonoid (-)-epicatechin modified the expression of genes related to atrophy or synthesis of proteins.

In addition, since obesity could lead to muscle atrophy, which can result in impaired muscle function, it would be relevant in future research to evaluate these signaling pathways in both direct obesity and offspring obese by programming fed postnatally with HFD in animals of different ages in order to search for markers of the progression of diseases such as sarcopenia obesity.

## Data Availability

No datasets were generated or analysed during the current study.
